# Fall Prevention in Adults With Cognitive Impairment: Systematic Review and Meta-Analysis

**DOI:** 10.1093/geroni/igab046.2603

**Published:** 2021-12-17

**Authors:** Megan Racey, Maureen Markle-Reid, Muhammad Usman Ali, Hélène Gagné, Susan Hunter, Jenny Ploeg, Richard Sztramko, Diana Sherifali

**Affiliations:** 1 McMaster University, Hamilton, Ontario, Canada; 2 Ontario Neurotrauma Foundation, Toronto, Ontario, Canada; 3 Western University, London, Ontario, Canada

## Abstract

Cognitive impairment increases an individual’s risk of falls due to the role cognition plays in gait control. Older adults with dementia fall 2-3 times more than cognitively healthy older adults and there is a lack of evidence for effective fall prevention interventions for community-dwelling cognitively impaired adults. We conducted a systematic review and meta-analysis to investigate the effectiveness of fall prevention interventions in improving falls, perceived risk of falls, gait, balance, and functional mobility. We searched 7 databases for interventions involving community-dwelling adults ≥50 years with mild to moderate cognitive impairment. Reviewers screened citations, extracted data, assessed risk of bias and certainty of evidence (GRADE). We performed a meta-analysis of 509 community-dwelling adults (mean age 67.5 to 84.0 years) with mild to moderate cognitive impairment from 12 randomized controlled trails (8 exercise interventions, 3 multifactorial, and 1 providing medication). Interventions had medium significant effects on perceived risk of falls (SMD -0.73 [-1.10, -0.36]), balance (SMD 0.66 [0.19, 1.12]), and timed up and go test (SMD -0.56 [-0.94, -0.17]) and small significant effects on gait speed and control (SMD 0.26 [0.08, 0.43]) with moderate certainty of evidence. There were no significant effects for falls. Sub-analysis showed that exercise and studies at low risk of bias remained significant for balance and perceived risk of falls. The effect of fall prevention interventions on falls remains unclear; exercise interventions are effective at addressing fall risk factors. However, high quality and longer studies with adequate sample sizes are needed to determine their effectiveness on falls.

